# Identification and analysis of urban functional area in Hangzhou based on OSM and POI data

**DOI:** 10.1371/journal.pone.0251988

**Published:** 2021-05-27

**Authors:** Ziyi Wang, Debin Ma, Dongqi Sun, Jingxiang Zhang

**Affiliations:** 1 School of Geography, Geomatics and Planning, Jiangsu Normal University, Xuzhou, China; 2 School of Architecture and Urban Planning, Nanjing University, Nanjing, China; 3 Institute of Geographic Sciences and Natural Resources Research, Chinese Academy of Science, Beijing, China; Northeastern University, CHINA

## Abstract

The accurate identification of urban functional areas is of great significance for optimizing urban spatial structure, rationally allocating spatial elements, and promoting the sustainable development of the city. This paper proposes a method to precisely identify urban functional areas by coupling Open Street Map (OSM) and Point of Interest (POI) data. It takes the central urban area of Hangzhou as a case study to analyze the spatial distribution characteristics of the functional areas. The results show that: (1) The central urban areas of Hangzhou are divided into 21 functional areas (6 single functional areas, 14 mixed functional areas and 1 comprehensive functional area). (2) The single functional areas and the mixed functional areas show the geographical distribution characteristics of the looping stratification, which means “Core-periphery” differentiation is obvious, and the comprehensive functional area is relatively scattered. (3) The mixed degree of regional function with ecological function and production function is low while comprehensive functional areas are usually associated with higher potential and vitality. (4) The identification results are in great agreement with the actual situation of Hangzhou central urban area, and the method is feasible. Therefore, this paper can provide a reference for urban development planning and management.

## Introduction

The functional area is the basic unit of urban planning, management and resource allocation. Urban functional areas are important geospatial attributes of urban land, in which people carry out various socio-economic activities, which are usually determined by two perspectives of land use type and human activities, including residential land, industrial land, commercial and business facilities land [1, 2, 7]. As the basic spatial unit of urban development, the concept of urban functional area originated from the "functionalism" planning idea established by the Athens Charter, which emphasizes the clear structure of the city, pays attention to the functional areas division and the purification of use [3]. Diversified urban functions bring convenience to the life, work, recreation, and communication of urban residents, which is the foundation and charm of urban sustainable development [4, 5]. In 2019, China’s urbanization rate exceeded 60%. With the accelerating process of urbanization, various elements gather and diffuse in different spaces of the city [6] and result in functional differentiation at different regional scales. In addition, unreasonable urban planning leads to such problems as a single functional urban structure, spatial differentiation, lack of affection and care and so on, which causes the loss of urban vitality and a series of urban environmental and social problems. Therefore, the accurate identification of space and social structure of the urban, the rational division of functional urban areas has become an important subject of current research. It is of great significance for coordinating the relationship between humans and land, optimizing the urban spatial strategy and improving the level of urban planning [7]. At the same time, the reasonable division of urban functional areas has certain guiding value for solving many urban diseases, such as traffic congestion, air pollution, waste of land resources, climate change and so on [8–10].

The traditional urban function zoning is mainly based on remote sensing images, land use data, panel data and so on [11]. Zhong et al. proposed a semantic allocation level multi-feature fusion strategy for high spatial resolution image scene classification which is based on the probabilistic topic model [12]. Zhang et al. proposed a complete hybrid scene decomposition system to decompose the high-resolution remote sensing images of Beijing and Zhuhai [13]. However, the traditional method of remote sensing can only classify urban functions based on the natural properties of the land. With the enhancement of the data acquisition capability, breaking the traditional idea of functional zoning, fully mining the urban social and cultural information contained in big data, and establishing the methodology system for identifying urban functional areas has become an innovation direction of urban geography, which especially in the context of information [14]. Liu collected 7-day taxi trajectories in Shanghai to study the temporal variation of boarding and alighting and its relationship with different land-use characteristics [15]. Pei et al. collected mobile phone data in Singapore to identify residents’ daily travel activities to reflect the social function of land use [16]. Jia et al. combined remote sensing image (RSI) of a large area with mobile phone positioning data (MPPD) and applied this framework to the center of Beijing [17]. Song et al. explored the use of Singapore’s parks based on the number of photos and visual content of the geographical location of the park (from Instagram and Flickr platforms) [18]. However, there are data acquisition difficulties and strong subjectivity problems in urban functional area identification based on remote sensing or survey data. The big data of travel or pictures can only be roughly divided into urban functional areas, which cannot accurately analyze the spatial structure of urban functions, nor can they provide the precise implementation to solve the problems of land use function [19, 20]. At the same time, these methods are limited by the number of users and the accuracy of the user footprint location, so the accuracy of the classification results is poor [21].

Compared with the above studies, the POI data is a kind of point-shaped geographic space big data of real geographic entities. The results of this research could finely characterize the dynamic and real-time nature of urban land functions, and reflect the diversity and mixed degree of various facilities [22, 23]. Zhai et al. improved the functional area identification framework by constructing the Place2vec model to capture POI geographic information [24]. Han et al. used POI data to identify single urban functional areas in Beijing [25]. Ran et al. conducted an in-depth analysis of the spatial pattern of the life service industry in Changsha based on POI data [26]. However, most of the present researches on the identification of urban functional areas which based on POI data are focused on the identification of the single function, and lack of in-depth division of mixed and comprehensive land-use functions. At the same time, in the division of the basic unit of functional area identification, present researches mainly divided into cells by grids, and lack of multi-functional identification of the actual land use units.

Therefore, this paper takes Open Street Map (OSM) road network data and POI data as the main data source, and proposes a method for accurate identification of urban functional areas based on kernel density estimation, functional area identification, mixed degree calculation and other technical means. Taking the main urban area of Hangzhou as a case, this paper further identifies urban single functional land, mixed and comprehensive functional land, and analyzes the distribution characteristics of the functional structure in the research area. The improvements of this study are shown as follows: 1) Taking the irregular grid formed by the road network as the research unit, the segmentation of urban functional areas is more reasonable. 2) The kernel density method reduces the noise effect of POI data, improves the recognition accuracy of mixed functional areas and weakens the discretization of results. 3) The recognition model considers the mixed functional areas and comprehensive functional areas to make the results more in line with the real situation. 4) The mixed degree calculation and accuracy verification of the identification results of urban functional areas are conducted to make the results more convincing. In terms of literature, it is expected to provide a typical case and research methods for the research of urban spatial structure in China driven by spatial big data. In practice, it provides the reference for urban managers to understand the urban spatial structure, accurately grasp the development status of urban functions, and formulate reasonable urban planning schemes.

## Study area and methods

### Study area: The central urban area of Hangzhou, China

**Fig 1:**
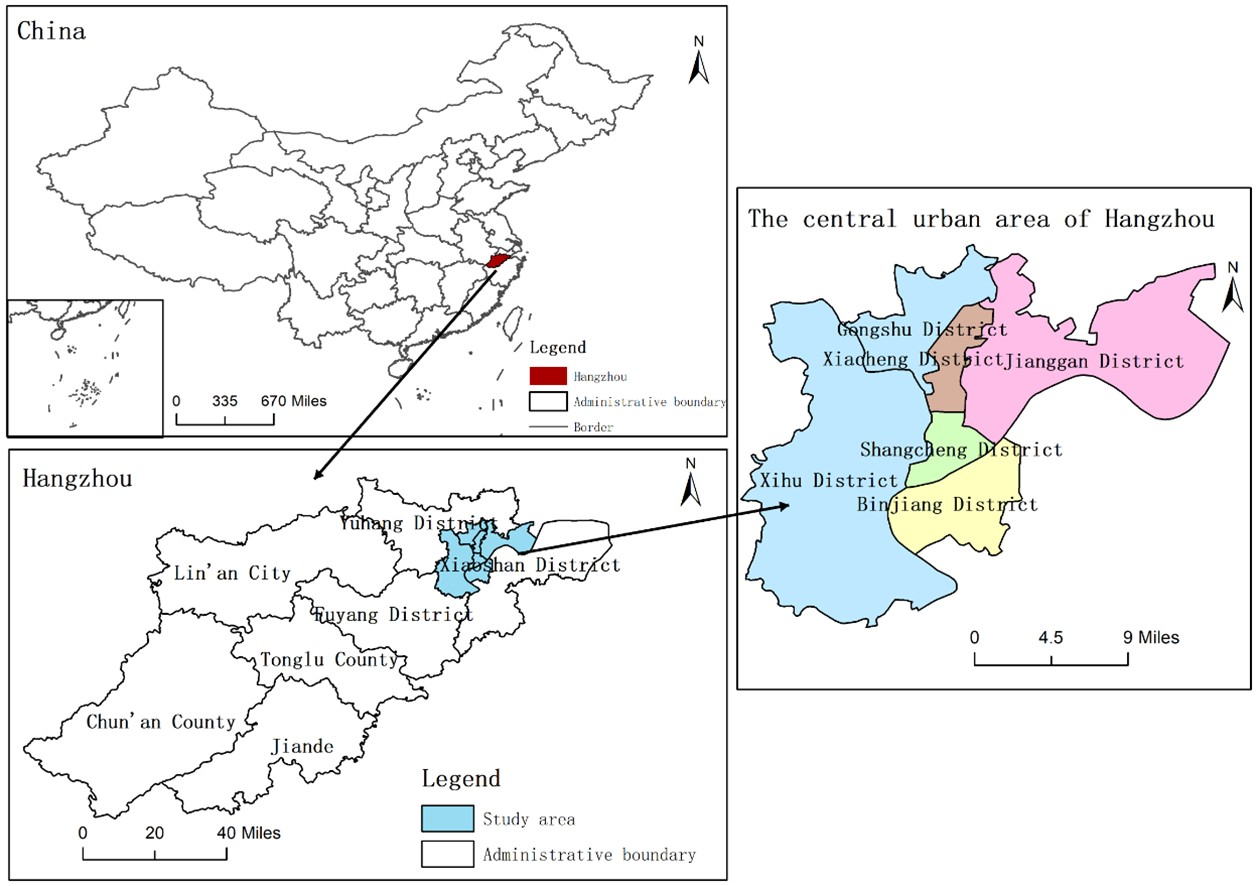
Map of the study area. The administrative division data are from the 1:250,000 basic geographic database provided the National Geomatics Center of China (http://www.ngcc.cn/ngcc/html/1//391/392/16114.html), these data are available free of charge.

Hangzhou, the capital city of Zhejiang Province, is located in the northern part of Zhejiang Province, the lower reaches of the Qiantang River, and the southern end of the Beijing-Hangzhou Grand Canal. The central geographical coordinates are E120°19’, N30°26’. The city governs 10 districts, 2 counties and 1 county-level city, with a total area of 16853.57km^2^. It is and one of the central cities in the Yangtze River Delta. In 2019, the city’s GDP reached 222.85 billion US dollars, with a permanent population of 10.36 million. The central urban areas are located in the northeast of Hangzhou, including Xihu District, Gongshu District, Shangcheng District, Binjiang District, Jianggan District, and Xiacheng District, with a total area of 706.27km^2^. The central urban areas are concentrated areas of business, population and services in Hangzhou. The urbanization process of Hangzhou is the epitome of China’s rapid development. At the same time, the urban development mode of Hangzhou, which takes into account science and technology, industry, humanities and ecology, represents the development direction of the future city. Moreover, the rapid change of urban functions of such cities brings challenges to urban management and operation. Therefore, this area is selected as the research area. An overview of the research area is shown in Fig 1.

### Data source and process

Before collecting the open source data required for the article, we have carefully examined all relevant platform service terms to ensure that our research is fully compliant with the agreement. The administrative division data is from the 1:250,000 basic geographic database provided the National Geomatics Center of China (http://www.ngcc.cn/ngcc/html/1//391/392/16114.html), the satellite map data comes from Auto Navi Map (https://www.amap.com/), and they all can be obtained free of charge. The urban road network data of Hangzhou comes from the Open Street Map (OSM) geographic data platform (https://www.openstreetmap.org/). OSM aims to provide users with free and easy-to-access digital map resources, which is currently the most popular spontaneous geographic information platform. We preprocessed the collected OSM road network data: select highways, railways, township main roads, urban main roads and roads, delete sidewalks, residential lanes, chaotic road network and other lines which are less important and do not affect the analysis results, delete duplicate lines. Filter to delete other redundant paths and broken paths and paths below 100 meters, and finally, fill the broken paths. POI (Point of Interest) data comes from AutoNavi Maps Open Platform (https://lbs.amap.com/). AutoNavi Maps is China’s leading provider of digital map content, navigation, and location services. This research uses the open API interface provided by AutoNavi Maps to obtain 168607 POI data of six districts in Hangzhou in December 2020, and each POI data contains attribute information such as geographical entity name, address, affiliation type, longitude, and latitude, administrative region and so on. There are many problems in the original POI data, such as various classifications, the repeated crossover between different classifications, classification errors, information missing, and inconsistent coordinate system. Moreover, some types of POI have low public awareness, such as public toilets, newspaper pavilions, and bus stations. These types of POI are not significant in the identification of functional areas and are not convenient for research and discussion. Therefore, it is necessary to unify the coordinate of POI data, clean the data, delete wrong POI points, delete repeated abnormal values, screen and delete low public awareness points, and reclassify them. According to the "Code for classification of urban land use and planning standards of development land" (GB_50137–2011) and combined with the actual conditions of Hangzhou, we divided POI into six types: residential, administration and public services, commercial and business facilities, industrial, green space, science and education [27] (Table 1).

**Table 1:** POI classification table.

Primary classification	Secondary classification	Three-level classification
**Residential**	Residential district, business residential district	Residential quarters, dormitories, villas, etc
**Commercial and business facilities**	Shopping services, life services, automobile services, catering services, accommodation services, leisure and entertainment, finance	Supermarkets, shopping centers, shopping streets, Chinese restaurants, foreign restaurants, star hotels, express hotels, cinemas, banks, ATM, credit cooperatives, etc
**Industrial**	Incorporated business, industrial and mining factory building, parks	Companies, factories, science and technology parks, industrial parks, etc
**Administration and public services**	Government agencies and social organizations, health care, public facilities	Government agencies, social organizations, public security organs, industrial and commercial tax authorities, hospitals, emergency centers, railway stations, airports, docks, etc
**Science and education**	Institutions of higher learning, vocational colleges, middle schools, primary schools, scientific and educational places, cultural media	Universities, middle schools, primary schools, kindergartens, vocational colleges, museums, libraries, art museums, archives, cultural palace, overseas study agencies, scientific research institutions, training institutions, etc
**Green space**	Tourist attractions, Park Square	Scenic spots, zoos, botanical gardens, parks, squares, churches, temples, etc

### Research methods

**Fig 2:**
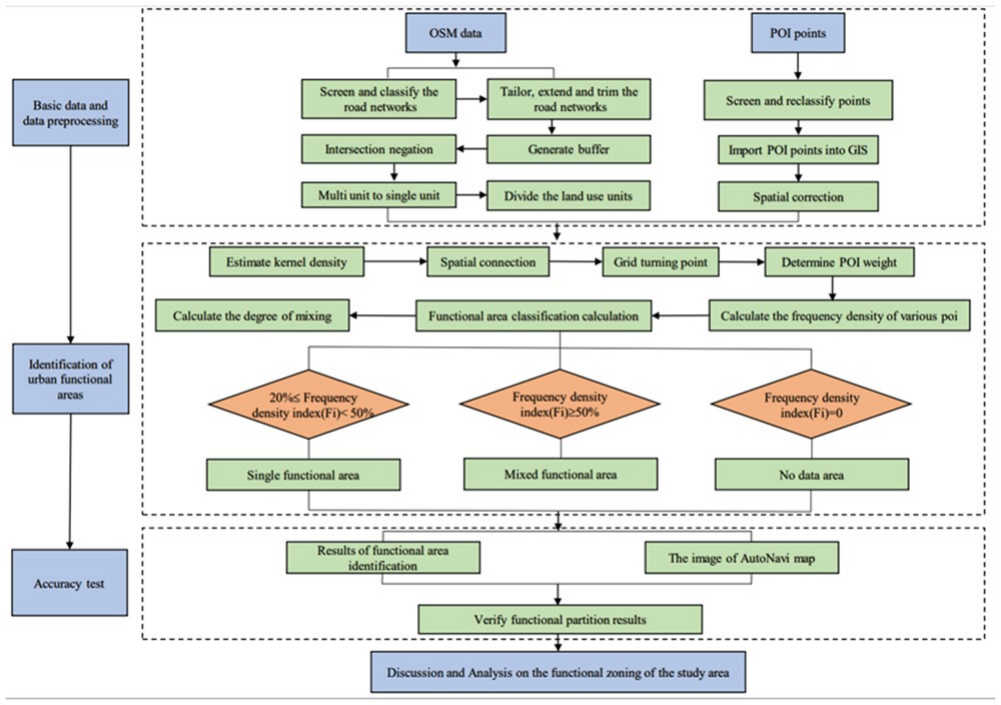
Flowchart of research.

The research idea of this paper is to use the OSM road network data to divide the city into different research units. Then, based on the POI data, the frequency density of different research units in the city is calculated by using the methods of kernel density and POI weight assignment. Finally, it is divided into different functional partitions according to the discriminant rules. The technical route is shown in Fig 2.

#### Research unit division based on OSM data

OSM refers to an open street map for the crowd. Ordinary users can collect and track a large amount of location or trajectory data through mobile terminals, and publish geographic information to the Internet with the help of an open map platform. Compared with traditional mapping spatial data, this kind of data has the advantages of real-time, fast update speed, and free access [28]. This paper collects road network data covering the research area, including five types: highways, arterial roads, first-class roads, second-class roads, and third-class roads. According to China’s "Code for classification of urban land use and planning standards of development land", the highways and arterial roads are the first level, the first-class roads and second-class roads are the second level, and the third-class roads are the third level, buffer zones of 40m, 20m, and 10m are generated respectively. When the irregular grid is generated, there are suspension points and independent roads are topologically modified to form closed units.

#### Kernel density estimation

The kernel density is based on the first law of geography, that is, the closer the objects are, the greater the density expansion value will be. This method is used to calculate the density of spatial points and line elements in their surrounding neighborhoods, and to simulate the density layout continuously. The kernel density value of each grid in the image reflects the layout characteristics of spatial elements. It is less affected by subjective factors, and the result has the advantages of gradual change and revealing local characteristics [29, 30]. In this research, the influence spreading of POI data is realized by using the kernel density method. The formula is defined as:

$$P_{i}=\frac{1}{n\pi R^{2}}\times \sum_{j=1}^{n}K_{j}{\left[1-\frac{D_{ij}^{2}}{R^{2}}\right]}^{2}$$ (1)

In the formula, *K*_*j*_ is the weight of the research object j, is the distance between the spatial point i and the research object j, R is the bandwidth of the selected regular area (*D*_*ij*_ < *R*), n is the number of research objects j within the bandwidth R.

The result shows that the choice of bandwidth R has a critical impact on the result of kernel density analysis [31, 32]. Generally, larger bandwidth is used to reflect the spatial variation of the global scale, and a smaller bandwidth reflects the spatial variation of the local scale. In this paper, the bandwidth R is adaptive by the optimal search bandwidth strategy of the ArcGIS10.5 platform, and the calculation formula is:

$$r=1\times min(SD,\sqrt{\frac{1}{11(2)}}\times D_{m})∕n^{-0.2}$$ (2)

$$SD=\sqrt{\sum_{i=1}^{n}{\left(x_{i}-\overset{-}{X}\right)}^{2}∕n+\sum_{i=1}^{n}{\left(y_{i}-\overset{-}{Y}\right)}^{2}∕n}$$ (3)

where *r* is the bandwidth, *SD* is the standard distance value, *D*_*m*_ is the median of the distance between the average center of the point and all points, *n* is the number of points, *x*_*i*_ and *y*_*i*_ are the coordinates of points, respectively, and $\overset{-}{X}$ and $\overset{-}{Y}$ are the average center coordinates of POI points, respectively.

The above formula is used for calculation in GIS to obtain the search radius of POI data of various functional areas. Among them, the residential area is 1095 m, the administration and public services area is 1102 m, the commercial and business facilities area is 745 m, the industrial area is 970 m, the green space is 1224 m, the science and education area is 1013 m, and the overall search radius of POI data is 837 m. Hinnerburg et al. researched the relationship between the bandwidth and the number of density-attractor and found that there are a certain number of bandwidth intervals that keep the density-attractor stable, and it is reasonable to choose the bandwidth in these intervals [33]. Based on this, the bandwidth interval is determined as [700m, 1300m]. Considering the unity of data analysis, 1000m bandwidth kernel density analysis can better describe the specific aggregation characteristics of various industries, which can meet the needs of urban spatial structure analysis [34, 35].

#### POI weight assignment

It is not only the distribution density of POI but also the size of geographical objects represented by POI and the degree of public recognition to affect the function of the plot. In this paper, the occupied area of POI points is selected as one of the weights (Table 2), and the average building area or occupied area of various POIs is determined by referring to the current format classification standard GB / T18106-2010 and the urban public service facility planning standard GB50442 (draft for comments), and then according to the data and the area value for grading and scoring (Table 3). According to the research of Xue and Zhao et al. [36, 37], public awareness is cited as another impact factor (Table 4). Then the weights of the two influencing factors are set to 1:9, 3:7, 5:5, 7:3 and 9:1, respectively. After the accuracy of each proportion of sampling verification, only the accuracy of the results with the weight of 5:5 reaches more than 80%. Therefore, in order to ensure the accuracy of the research, we set the weight of the two influencing factors as 5:5. After superposing the weights of different POI data in different land-use types, the corresponding weights of different land-use types are given. The full score of the two factors is 100, and the total score after weighting is 100. For example, if the general area score of a POI point is 50 and the public awareness is 0.7 (the percentage is 70), then the weight assignment result of this POI point is 60. Based on these, the corresponding weights of each land type are: science and education 65, administration and public services 50, residential 30, commercial and business facilities 25, industrial 70, and green space 80.

**Table 2:** Functional classification of POI and reference value of coverage area.

Primary classification	Secondary classification	Reference area (ha)	Remarks
**Residential**	Residential district	3	
	Business residential district	0.1	
	Villas	1	
	Dormitories	0.1	
**Commercial and business facilities**	Shopping services	5/3/0.3/0.01	Shopping services include shopping malls, shopping malls, supermarkets and convenience stores
	Life services	0.02	
	Automobile services	0.05/0.3	Automobile service is divided into automobile maintenance or automobile 4S shop
	Catering services	0.02/0.1	The catering service is divided into general restaurants or high-end restaurants
	Accommodation services	0.08	
	Leisure and entertainment	0.05	
	Financial insurance	0.02/0.5	Financial services are divided into financial and insurance stores, financial and insurance companies
**Industrial**	Incorporated business	0.4	
	Industrial and mining factory building	1	
	Parks	6	
**Administration and public services**	Government agencies and social organizations	1	
	Health care	5/1.5/0.1	Medical and health care is divided into comprehensive type, specialized type, clinic and pharmacy
	Public facilities	0.02	
**Science and education**	Institutions of higher learning	10	
	Scientific research units	0.2	
	Primary and secondary schools	2	
	Other	0.01	
**Green space**	Tourist attractions	2	
	Park Square	3	

**Table 3:** The evaluation of different area.

Area(m^2^)	0–100	100–1000	1000–3000	3000–5000	5000–10000	>10000
**Score**	1	10	30	50	80	100

**Table 4:** Public awareness of POIs of every category.

Categories	Public awareness	Categories	Public awareness
Residential	0.01	Science and education	0.6706
Business residential district	0.3057	Tourist attractions	0.8245
Business residential district-related	0.01	Park Square	0.6548
Catering services	0.5562	Health care	0.5069
Shopping services	0.8146	Government office	0.355
Accommodation services	0.5562	Incorporated business	0.3057
Leisure and entertainment	0.501	Financial insurance	0.3057

### Urban functional area identification

According to the weight calculation of each POI, the frequency density of each POI in the land use unit is calculated, which can be used as the basis of functional zoning [3]. The calculation formula is as follow:

$$F_{i}=W_{i}\times d_{i}/\sum_{j=1}^{6}\left(W_{j}Xd_{j}\right)\times 100\%$$ (4)

Where *F*_*i*_, *d*_*i*_, and *W*_*i*_ are the frequency density, sum of nuclear density, weight of the POI of class I in the unit, respectively. Due to the complex and diverse urban functional structure, there are both single-use functional areas and areas with two or more mixed functions. Therefore, this paper further compares the frequency density of POI in the functional area: When the frequency density of POI in the unit is more than or equal to 50%, the functional area is a single function area. When the two kinds of values of higher frequency density of POI in the unit are between 20% and 50%, the unit is defined as a mixed functional area with two types of POI. When the frequency density of all kinds of POI in the unit is 0, it is defined as no data area, and the rest of the unit is the comprehensive functional area.

#### Mixed degree

According to the mixed index of land use, the mixed degree of functional areas in the research area is further evaluated [38]. The formula is as follow:

$$D=-\sum_{i=1}^{n}\left(p_{i}{lnp}_{i}\right)$$ (5)

Where D is the calculation result of the mixing degree, *p*_*i*_ is the proportion of class I POI kernel density value to the total value of each class of POI kernel density in the functional area unit, and n is the number of POI classes. If the kernel density of some POI in the unit is 0, it does not participate in the calculation.

## Results

### Results of urban functional area identification

**Fig 3:**
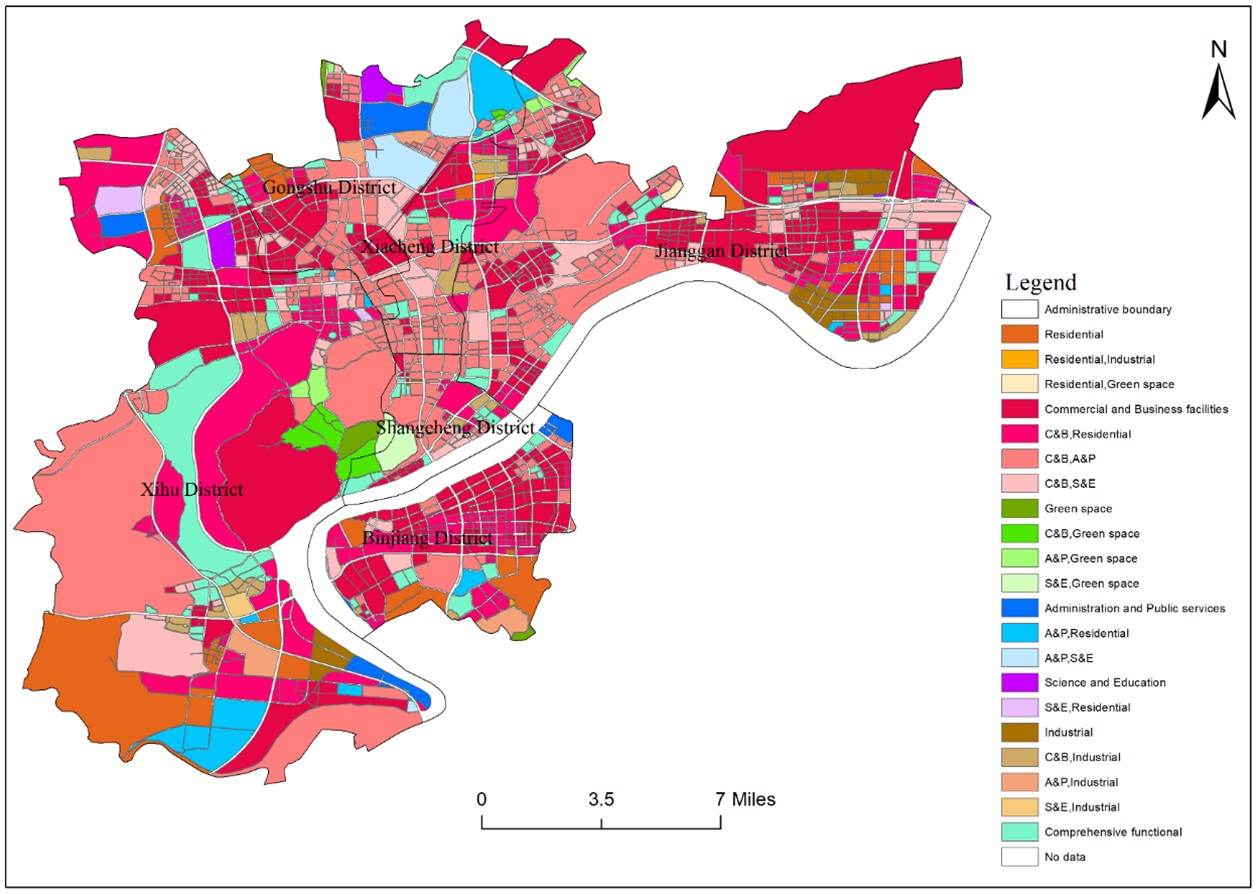
Recognition results of functional areas in the central city area of Hangzhou.

Through OSM network grading and buffer operation, the central urban area of Hangzhou is divided into 1542 block units. After calculating the weight and frequency density of various POI in each block unit, the identification result of functional areas in the Hangzhou central district is obtained (Fig 3). It can be seen from Fig 3 that there are 21 types of functional areas in downtown Hangzhou, including 6 types of single functional areas, 14 types of mixed functional areas, and 1 comprehensive functional area. Single functional areas have commercial and business facilities land (422 blocks in total, with a total area of 153.032 km^2^), residential land (76 blocks in total, with a total area of 48.128 km^2^), administration and public services land (11 blocks in total, with a total area of 8.703 km^2^), green space land (5 blocks in total, with a total area of 2.369 km^2^), industrial land (35 blocks in total, with a total area of 7.957 km^2^), science and education land (4 blocks in total, with a total area of 2.989 km^2^). The mixed functional areas include the combination of commercial and business facilities land, and administration and public services land (350 blocks in total, with a total area of 144.794km^2^), the combination of commercial and business facilities land, and residential land (234 blocks in total, with a total area of 83.733km^2^), the combination of commercial and business facilities land, science and education land (136 blocks in total, with a total area of 34.917km^2^), the combination of commercial and industrial facilities land (39 blocks in total, with a total area of 10.678km^2^), the combination of commercial and business facilities land, and green space land(7 blocks in total, with a total area of 5.466km^2^), the combination of administration and public services land, and residential land (14 blocks in total, with a total area of 15.317km^2^), the combination of administration and public services and green space land (7 blocks in total, with a total area of 2.714km^2^), the combination of administration and public services and industrial land (6 blocks in total, with a total area of 6.084km^2^), the combination of administration and public services land, and science and education land (4 blocks in total, with a total area of 8.618km^2^), the combination of industrial and residential land (2 blocks in total, with a total area of 0.258km^2^), the combination of residential and green space land (1 block in total, with a total area of 0.411km^2^), the combination of science and education, and residential land (4 blocks in total, with a total area of 2.838km^2^), the combination of science and education, and industrial land (1 block in total, with a total area of 0.961km^2^), the combination of science and education, and green space land (2 blocks in total, with a total area of 2.938km^2^). There are 103 comprehensive functional areas, covering an area of 39.801km^2^. The remaining units are no data areas (79 blocks in total, with a total area of 0.333km^2^). Because of the small statistics of various POIs in this block, the kernel density value is small too.

### Spatial distribution of different functional areas

#### Single functional area

**Fig 4:**
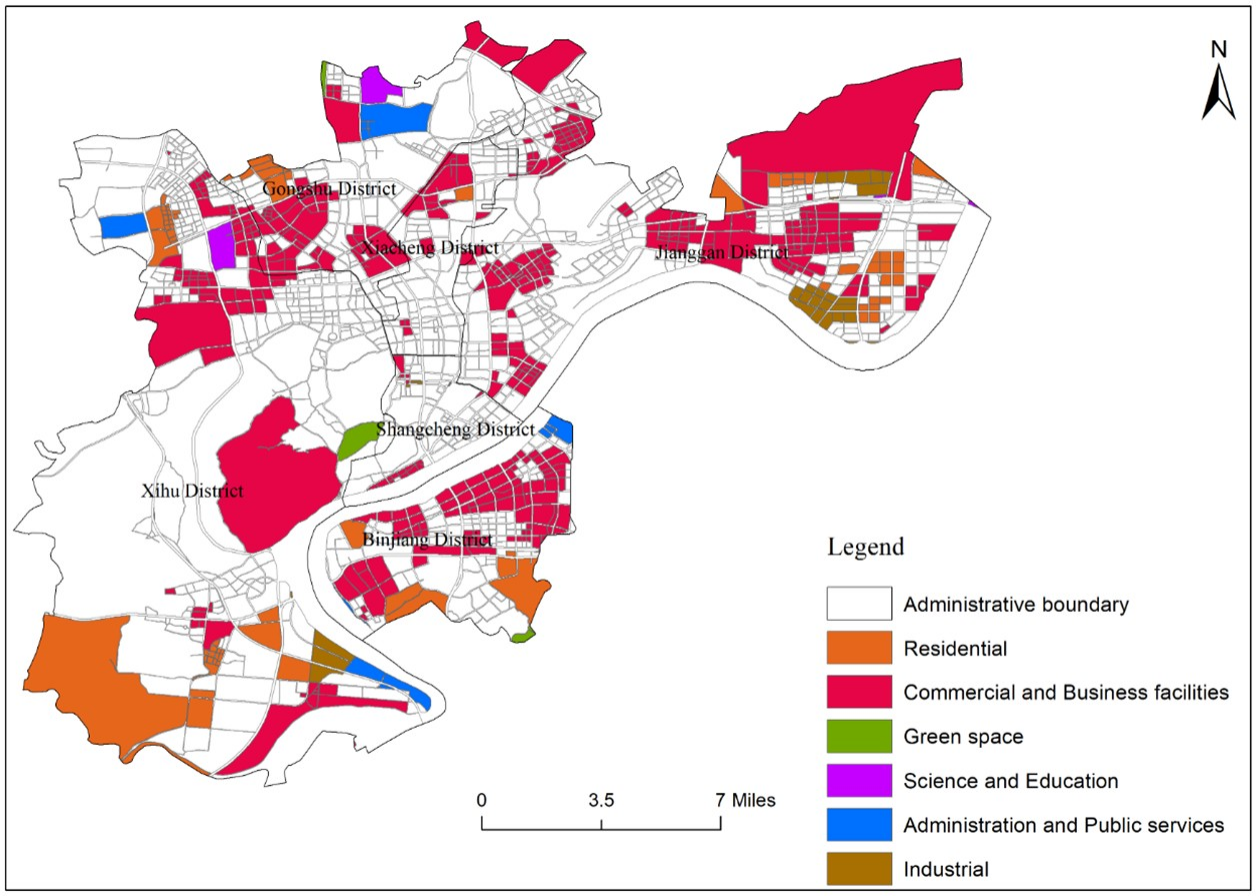
Distribution map of single functional areas in the central city area of Hangzhou.

The single functional areas in the central urban area of Hangzhou are mainly commercial and business facilities land and residential land (Fig 4). Among them, the number of commercial and business facilities land plots is the largest, accounting for 76% of the total land plots in the single functional area, and 27% of the total land plots in the research area. And the total area of commercial and business facilities land plots is the largest, accounting for 69% of the total land plots in the single functional area, and 26% of the total land plots in the research area. Commercial and business facilities land is distributed in a wide range, but there are large differences between regions, showing the characteristics of more periphery and less center. The number of residential land accounts for 13.7% and 4.9% of the single functional areas and the total of research areas, and the area accounts for 24% and 9%, respectively. Residential land’s spatial distribution is similar to that of commercial and business facilities land, showing the characteristics of more periphery and less center, residential land mainly distributed in the north and south of Xihu District, the east of Jianggan District, and the southern edge of Binjiang District. Meanwhile, the number of industrial land accounts for 6% of the single functional area and 2% of the total research area, with the area accounting for 1% and 0.4% respectively. Industrial land is mainly distributed along the main traffic arteries. The administration and public services land, science and education land, and green space have small areas and single distribution because there are many other mixed functions and the recognition rate is low.

#### Mixed functional area and comprehensive functional area

**Fig 5:**
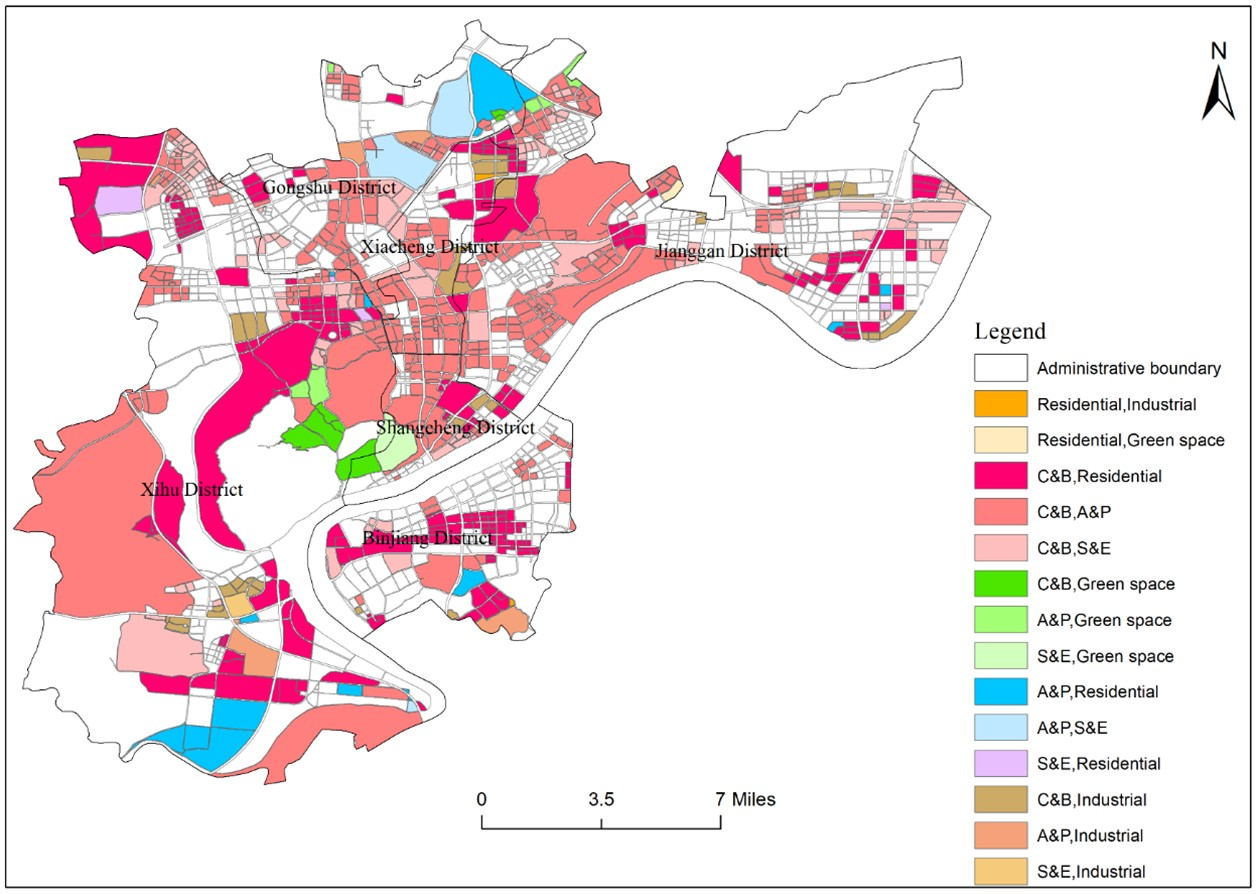
Distribution map of mixed functional areas in the central city area of Hangzhou.

**Fig 6:**
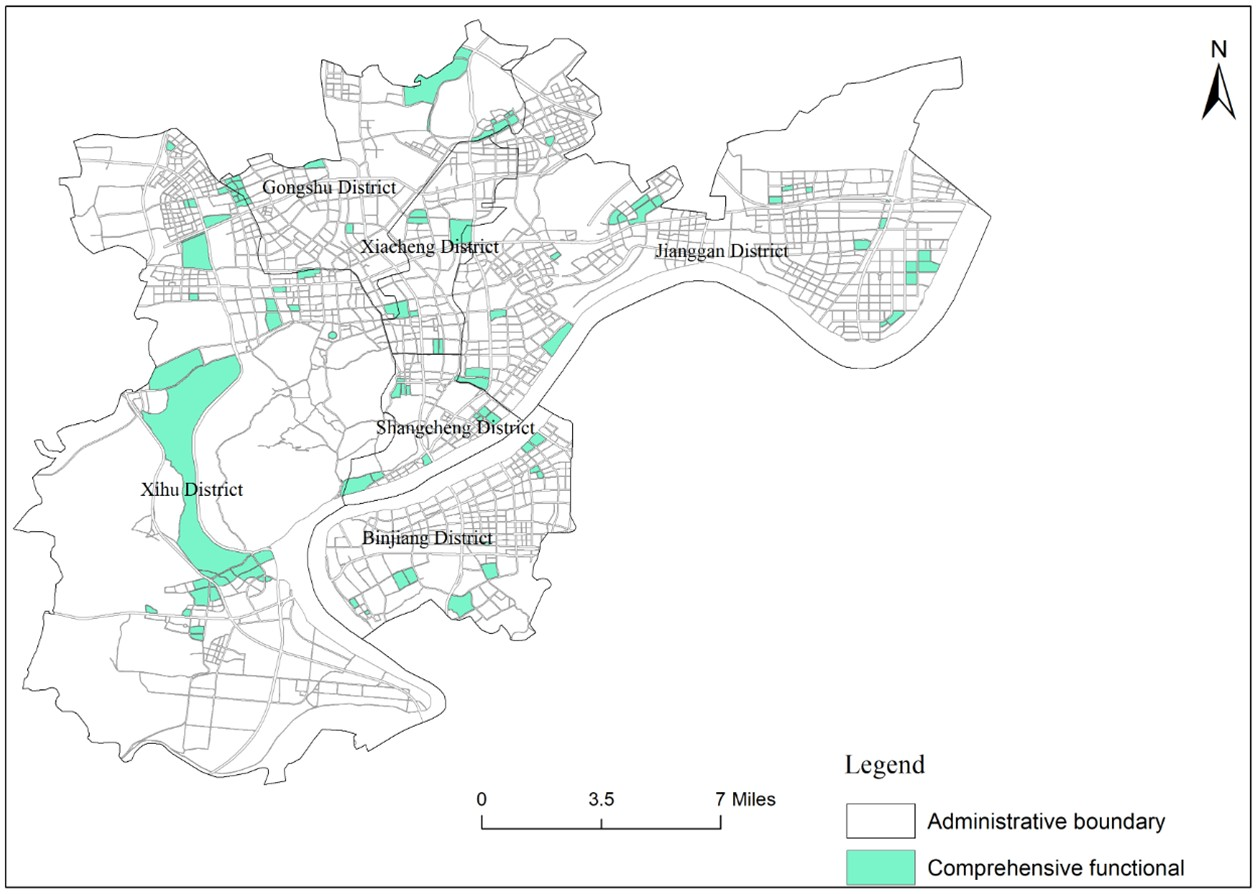
Distribution map of comprehensive functional areas in the central city area of Hangzhou.

Mixed functional areas in the downtown area of Hangzhou are widely distributed (Fig 5), with an area of 319.767km^2^, accounting for 55% of the total area of the research area, which indicates that the mixed degree of urban functions in the downtown area of Hangzhou is relatively high. Mixed functional areas are mainly the combination of commercial and business facilities land and administration and public services land, which occupy the largest area, accounting for 25% of the total research area. In terms of spatial distribution, the central part is more and concentrated, while the periphery is less and scattered. Specifically, they are primarily concentrated in Shangcheng District and Xiacheng District, the south of Gongshu District, the east and west of the Xihu District, and the west of Jianggan District. Next is the combination of commercial and business facilities land and residential land, accounting for 14% of the total research area, intersperses in various functional areas. The number of other mixed functional areas is 223, accounting for 14% of the total area of the research area, and the area of other mixed functional areas accounted for 16% of the total area of the research area. The layout of other mixed functional areas is more dispersed. Among them, the number of the combination of residential and green space land and the combination of science and education and the industry land are the least, with 1 each. The area of the combination of industrial and residential land is the smallest, accounting for only 0.04% of the total area of the research area. The overall layout of the comprehensive functional urban areas in the central city area of Hangzhou is scattered (Fig 6), accounting for 6.7% of the total research area, and the area of comprehensive functional urban areas accounted for 6.8% of the total functional areas. So it is clear that the overall layout of integrated functional urban areas in the center is in the state of multi-point flowering, distributed around all kinds of single functional areas and mixed functional areas.

#### Distribution characteristics of mixed utilization degree

**Fig 7:**
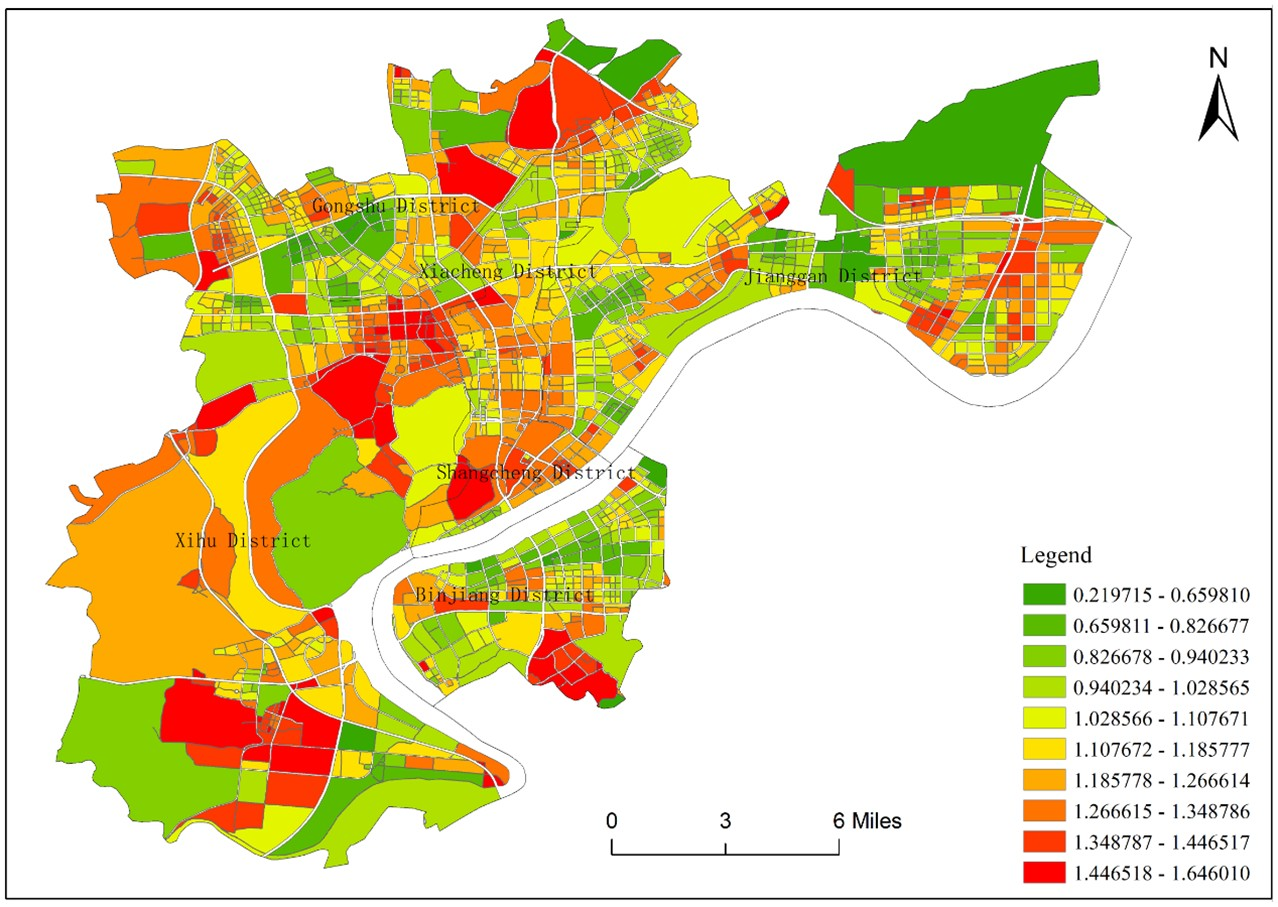
Distribution map of the mixed degree of functional areas in the central urban area of Hangzhou.

According to the distribution map of the mixed degree of functional areas (Fig 7), it can be clearly seen that the mixed-use degree of land use in the central urban area of Hangzhou has obvious regional differentiation. Those with a high mixed degree are mixed functional area and comprehensive functional area, and those with low mixing degree are the single functional area. The high-value areas are mainly distributed in the south of Xihu District and Binjiang District, the middle of Shangcheng District and Xiacheng District, the west of the Gongshu District, and the riverside area of Jianggan District. The low degree mixed areas are mainly distributed in the west of Xihu District, the north of Binjiang District, the south of Shangcheng District and Gongshu District, and the north of Xiacheng District and Jianggan District. In each administrative region, there are high-value areas and low-value areas of mixing degree, and the distribution is more even.

### Validation of results

**Fig 8:**
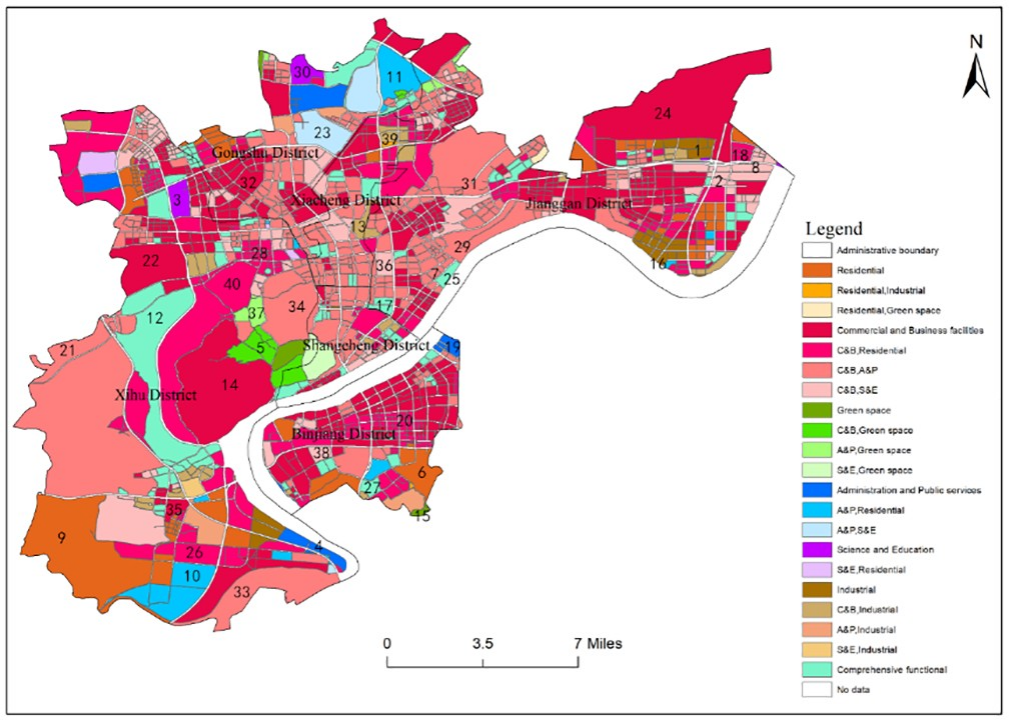
Sampling inspection of urban functional areas in central city area of Hangzhou.

In order to verify the accuracy of the functional area identification results, this paper draws on the research of Ding and Kang [39, 40]. In this paper, 40 units of functional area are selected randomly (Fig 8), and the real attributes of the units of functional area are judged according to AutoNavi map, and the accuracy of the identification results is evaluated by the method of scoring conformity degree. The full score is 3, that is, complete compliance, 0 is complete non-compliance. If the single functional area is identified as a mixed functional area of one type, it is 2, and the rest is 1. If the mixed functional area is identified as a single functional area that contains one function of this mixed functional area or a mixed functional area which contains one function of this mixed functional area, it is 2, and the rest is 1.If the comprehensive functional area is identified as a mixed functional area, it is 2, If the comprehensive functional area is identified as a single functional area, it is 1.

The accuracy calculation formula is:

$$a=\frac{\sum_{i=1}^{n}x_{i}}{\sum_{i=1}^{n}X_{i}}\times 100\%$$ (6)

In the formula, *n* is the sampling number and *X*_*i*_ is the sum of all samples with full precision, *x*_*i*_ is the actual score of sample accuracy.

The evaluation and calculation of the accuracy of functional zoning is shown in Table 5. It can be seen that the overall accuracy of urban functional area identification in the central urban area of Hangzhou reaches 82%, indicating that this research can effectively identify the urban functional areas and have certain accuracy.

**Table 5:** Accuracy evaluation of urban functional area.

Functional area number	Real attributes	Identified attributes	Accuracy score
1	Industrial	Industrial	3
2	C&B, S&E	C&B, S&E	3
3	Science and Education	Science and Education	3
4	Administration and Public services	Administration and Public services	3
5	C&B, Green space	C&B, Green space	3
6	Comprehensive functional	Residential	1
7	Science and Education	C&B, S&E	2
8	Science and Education	C&B, S&E	2
9	Residential	Residential	3
10	A&P, Residential	A&P, Residential	3
11	Green space	A&P, Residential	0
12	Comprehensive functional	Comprehensive functional	3
13	C&B, Industrial	C&B, Industrial	3
14	C&B, Green space	Commercial and Business facilities	2
15	Green space	Green space	3
16	Industrial	Industrial	3
17	Comprehensive functional	Comprehensive functional	3
18	C&B, Residential	C&B, Residential	3
19	Administration and Public services	Administration and Public services	3
20	C&B, Residential	C&B, Residential	3
21	C&B, S&E	C&B, A&P	2
22	C&B, Green space	Commercial and Business facilities	2
23	A&P, Industrial	A&P, S&E	2
24	C&B, Industrial	Commercial and Business facilities	2
25	Comprehensive functional	Comprehensive functional	3
26	C&B, Residential	C&B, Residential	3
27	Comprehensive functional	Comprehensive functional	3
28	C&B, Residential	C&B, Residential	3
29	Comprehensive functional	C&B, A&P	2
30	Comprehensive functional	Science and Education	1
31	C&B, Industrial	C&B, A&P	2
32	Comprehensive functional	Commercial and Business facilities	1
33	C&B, A&P	C&B, A&P	3
34	A&P, Green space	C&B, A&P	2
35	Commercial and Business facilities	Commercial and Business facilities	3
36	C&B, S&E	C&B, S&E	3
37	A&P, Green space	A&P, Green space	3
38	C&B, S&E	C&B, S&E	3
39	C&B, Industrial	C&B, Industrial	3
40	S&E, Green space	C&B, Residential	0

## Discussion and suggestions

### Method feasibility analysis

In the past, the basic data of urban functional area identification were mainly census data and government planning text. These data have some limitations (high acquisition cost, difficult management, poor timeliness, etc.), making research in this field not detailed enough. With the development and popularization of computer technology, remote sensing technology and big data are applied in the research of urban functional area recognition, but there are still problems such as tedious classification methods and the result precision cannot be guaranteed. Based on the kernel density estimation algorithm, this paper proposes an urban functional area identification method that combines OSM and POI data. It has a high reference value for the analysis of urban overall patterns and spatial optimization. At the same time, the availability of OSM and POI data makes this method can be easily used in other cities.

Firstly, POI data can provide a spatial location and attribute information of various urban facilities. Real-time and efficient data acquisition methods simplify the cost and uncertainty of traditional identification methods such as remote sensing, field research and questionnaire survey. The application of big data breaks through the problems of poor timeliness and a small sample of traditional data, improves the accuracy of urban functional area identification, and is convenient for larger-scale research.

Then, the OSM road network is used to generate road space, and the research area is divided into independent block units so that the segmentation of urban functional areas is more reasonable. The kernel density estimation method is used to realize the influence diffusion of POI in the adjacent location, weaken the discrete phenomenon of POI points, and identify the functional areas through the optimal bandwidth selection. The results are basically consistent with the actual situation. Through the calculation of the mixed index of urban functional areas, it is convenient to express the mixed degree of land use in the central urban area of Hangzhou. Although in the process of urban planning and construction, urban land use layout planning emphasizes functional zoning, mixed land use planning should also be adhered to promote economic development and facilitate the lives of residents.

Finally, compare the identified functional areas with the image of AMap, the verification of the results shows that the method has a high degree of recognition for urban functional areas, which can make up for the disadvantages of the traditional method and has certain feasibility. It can support the spatial layout research of urban functional areas, so that the decision-making organs can find the shortcomings of the existing urban planning, and can coordinate the development direction of the existing planning functional areas, improve the vitality of urban space, and carry more human activities.

However, this method also has some shortcomings. For example, the verification of the results is only carried out through the comparison of typical regions, and there is no true value as a comparison. Therefore, it is hard to determine the accurate identification accuracy of this method. In addition, this method is based on the OSM road network to divide urban units, and the basic road network data have the problems of low density and the lack of data in suburban and rural areas, which leads to the excessive division of functional units in such areas, resulting in poor local recognition results.

### Current situation analysis

The urban functional area projects each element of the city in space and forms a closely related organism. The identification of Hangzhou urban functional areas based on big data is not only beneficial to the adjustment of urban spatial layout and the creation of good conditions for the development of urban economy and society but also can improve the efficiency of urban land use [41] through the rationalization of urban functional zoning, so as to ensure the effective implementation of urban sustainable development strategy [42, 43]. According to the “Results” part, we have the following judgments:

#### (1) The urban functional pattern of "single-mixed-comprehensive" coexistence

On the whole, the single functional areas are mainly distributed in the periphery of the city. For example, the single residential areas are mainly distributed in the north and south of West Lake District, the east of Jianggan District, and the southern edge of the Binjiang District. The single industrial areas are mainly distributed in the eastern part of the Jianggan District and the southern part of the Xihu District. The single areas for science and education are mainly distributed in the northern part of the main urban area of Hangzhou. In general, the more single functional land, the more it affects the mobility of the city [44]. For example, the time cost of residents leaving the residential area to reach two or more other functional areas is relatively higher. From the perspective of sustainable development, the simplification of functions will not only affect the "mobility" of the city but also directly reduce the attractiveness and vitality of the city [45]. The existence of single-use areas is related to policies, history, and land use cost. For example, the cost of land use in central areas may be more than ten times that in the suburbs. Therefore, large scales of land development are often developed in the suburbs [15]. At the same time, the development area policies with Chinese characteristics will also lead to the emergence of large-scale single functional areas in the suburbs.

The mixed functional areas are distributed in the whole research area and nested with single functional areas, which indicates the rationality of the spatial structure of the main urban area of Hangzhou. For example, residential land forms residential-industrial zones around enterprises, commercial-residential lands around businesses, and public service-residential lands around public services. From the perspective of different administrative regions, each administrative region has an area with a higher degree of mixed, as well as an area with a lower degree of mixed. In line with the law of urban growth and development in urban areas, it revolves around multiple economic activity centers [46, 47]. However, most of the highly mixed areas are not centers of economic activity and do not have highly mixed urban functions around traffic stations. This is mainly due to the rigid system of land use and the consolidation of management models in China [48, 49].

#### (2) Comprehensive functional areas bring more vitality to the city

The comprehensive functional area means that there are three or more urban functions in the plot, reflecting the organic integration of different urban functions. For example, the southernmost comprehensive functional area of Binjiang District is the location of China Network Writers’ Village and the core for the development of China’s network literature industry. Meanwhile, the high-quality environment and supporting facilities attract more writers. Similarly, in the easternmost comprehensive functional area of Jianggan District, there are enterprise platforms with functions of scientific research and industrial incubation, such as ABO Biopharmaceuticals (Hangzhou) Co., Ltd., Sanhua Academia Sinica, as well as high-grade residential areas, the supporting facilities are relatively complete. These regions are very typical innovation areas in Hangzhou, and the mixing of functions is also the most prominent feature of high-quality urban innovation space [50, 51].

#### (3) Low mixed degree of regional function with ecological function and production function

In the research area, the mixed degree of some areas is deliberately controlled, mainly in the areas that undertake ecological functions, such as the Qiantang River, Xixi Wetland, West Lake Scenic Area, and the areas that undertake production functions, such as Zhejiang Qiosi Farm in Jianggan District. This is because the strategy of ecological civilization and the protection of cultivated land both are China’s basic state policies [52], which require strict restrictions on the development of the ecological area and cultivation area. A high degree of mixed often means diversified and high-intensity land development. Therefore, the existence of such low-mixed land use for urban development strategy is of great significance.

### Suggestions

The rapid evolution of cities and the promotion of China’s urbanization policy have brought many new challenges to urban planning and management. The spatial division of urban functional areas and urban zoning management of the city could provide a new idea for planners and managers to a certain extent. The government could plan different functional areas of the city according to the social, economic and natural geographical conditions, and realize the change from the traditional " unitary and extensive " management mode to the modern " diversified and refined " management mode [53, 54].

Based on the above analysis of the comprehensive judgment of the current situation, we believe that the current urban functions of the central city area of Hangzhou are becoming diversified and individualized. However, with the continuous evolution of urban spatial structure, there are many potential problems [55]. First of all, the single functional area is still more, leading to the separation of working and housing, the separation of school and residence, and heavy traffic pressure in the morning rush and evening rush. Secondly, the less comprehensive function area and insufficient space for diversified urban functions, which will bring the problem of insufficient vitality and attraction of the city. Finally, the over-strict control of land function results in the restriction of the diversified allocation of ecological and productive land, which has a great inconvenience to the surrounding residents.

Therefore, in the future, the urban land use function of the central urban area of Hangzhou should be optimized from the following aspects: (1) Developing mixed-use areas based on actual needs, guiding the optimization of urban mixed areas to improve the efficiency of land use. (2) Reducing single-use land types and promoting the transition from single-use land to mixed-use land. (3) Adhering to the construction of the multi-center distribution pattern of urban spatial structure and promote the fair distribution of social-spatial elements [56]. (4) While adhering to the strict system of cultivated land protection and ecological protection, we should also provide diversified infrastructure to serve the neighboring residents.

## Conclusions and deficiencies

This article takes the central urban area of Hangzhou as the research area and proposes a method of city functional area identification based on the kernel density estimation algorithm that integrates OSM road network and POI data: Firstly, the OSM network divides the urban area into research units with the same social and economic functions. The influence diffusion of POI facilities is realized based on the kernel density estimation algorithm, which weakens the discretization of POI. At the same time, kernel density bandwidth and POI weight are set to identify the functional urban areas and projected to different plots. Finally, the land-use mixed index is calculated. The results show that: (1) There are three types of urban functions in the central urban area of Hangzhou: single, mixed, and comprehensive. The central urban areas of Hangzhou are divided into 21 functional areas and the commercial function land is the dominant function of the research area. (2) The single functional areas and the mixed functional areas show the geographical distribution characteristics of the looping stratification, which means the “Core-periphery” differentiation is obvious. Mixed functional areas are widely distributed, but the areas with ecological and production functions have a low degree of mixed. Comprehensive functional areas are widely distributed and with higher potential and vitality. (3) The results of identification are in good consistent with the actual situation of the central urban area of Hangzhou. Therefore, the kernel density weighting method which considered public awareness and general area has high accuracy and feasibility, which can provide a reference for urban development planning and management. (4) The real-time and high efficiency of OSM and POI data acquisition methods and the scientific accuracy of big data application make the method of identifying urban functional area which combined OSM and POI data available. This method can be easily used in the identification of other urban regional functional areas to help people to understand the urban spatial structure more intuitively. And this method has high potential in assisting government departments and researchers in the scientific analysis of urban planning and land use.

There are some deficiencies of this paper: Because of the data defect of POI, the identification ability of areas such as small building density, unused land in cities and farmland is weak. At the same time, the integrity of POI data, the rationality of data cleaning and the accuracy of reclassification need further research. In view of the defects of the results test in research methods, a variety of verification methods can be added to future research for a horizontal comparison of results. Finally, due to the lack of historical POI, the evolution characteristics of urban functional areas cannot be identified. So combining mobile signaling data, sharing vehicle (bicycle) data, social media big data and other multi-source data to analyze the evolutionary logic and driving mechanism of urban functional areas will be the direction of future research.
